# Micro-RNAs: Crossroads between the Exposure to Environmental Particulate Pollution and the Obstructive Pulmonary Disease

**DOI:** 10.3390/ijms21197221

**Published:** 2020-09-30

**Authors:** Mauro Finicelli, Tiziana Squillaro, Umberto Galderisi, Gianfranco Peluso

**Affiliations:** 1 Research Institute on Terrestrial Ecosystems (IRET), National Research Council of Italy (CNR), via Pietro Castellino 111, 80131 Naples, Italy; 2 Department of Experimental Medicine, Division of Molecular Biology, Biotechnology and Histology, University of Campania “Luigi Vanvitelli”, via Santa Maria di Costantinopoli 16, 80138 Naples, Italy; tiziana.squillaro@unicampania.it (T.S.); umberto.galderisi@unicampania.it (U.G.)

**Keywords:** particulate matter, pollution, miRNA, heavy metals, COPD

## Abstract

Environmental pollution has reached a global echo and represents a serious problem for human health. Air pollution encompasses a set of hazardous substances, such as particulate matter and heavy metals (e.g., cadmium, lead, and arsenic), and has a strong impact on the environment by affecting groundwater, soil, and air. An adaptive response to environmental cues is essential for human survival, which is associated with the induction of adaptive phenotypes. The epigenetic mechanisms regulating the expression patterns of several genes are promising candidates to provide mechanistic and prognostic insights into this. Micro-RNAs (miRNAs) fulfil these features given their ability to respond to environmental factors and their critical role in determining phenotypes. These molecules are present in extracellular fluids, and their expression patterns are organ-, tissue-, or cell-specific. Moreover, the experimental settings for their quantitative and qualitative analysis are robust, standardized, and inexpensive. In this review, we provide an update on the role of miRNAs as suitable tools for understanding the mechanisms behind the physiopathological response to toxicants and the prognostic value of their expression pattern associable with specific exposures. We look at the mechanistic evidence associable to the role of miRNAs in the processes leading to environmental-induced pulmonary disease (i.e., chronic obstructive pulmonary disease).

## 1. Introduction

Environmental pollution has emerged as the most serious problem jeopardizing public health in the 21th century [1,2,3]. Increased air pollution, global warming, widespread use of toxicants, and radical changes in lifestyle are major concerns affecting modern society [4]. In particular, air pollution is global and recognized as one of the principal drivers of climate change [5]. The scientific community relies on environmental research to address these issues, evaluating and monitoring the potentially hazardous effects of toxicants on human health. Ubiquitous exposure to a plethora of pollutants is a given in modern life. This could be causative for alterations in many physiological processes and could lead to disease and abnormalities in exposed individuals [6]. An increased risk of congenital anomalies and perinatal mortality due to maternal exposure during pregnancy in polluted areas has been also documented [7].

Air pollution contains a complex mixture of harmful substances, especially particulate matter and heavy metals (e.g., cadmium, lead, and arsenic). The small size of these particles allows them to penetrate into the lung tissue, deposit into the alveoli, and even reach the blood stream [8]. Oxidative stress and immune response are the principal mechanisms activated by exposure to these elements. Such a situation can trigger a cascade of events, putting organisms at risk of experiencing adverse outcomes such as cardiovascular diseases, cancer, and especially, respiratory diseases [9,10,11,12].

The adaptive response to environmental cues is essential for human survival, and it is associated with the induction of adaptive phenotypes [13]. The epigenetic mechanisms controlling gene expression appear as a suitable model to provide mechanistic and prognostic insights into this. Among them, micro RNAs (miRNAs), a class of small non-coding RNAs, have emerged as intriguing candidates in understanding the changes in gene expression from exposure to environmental cues. These molecules mediate post-transcriptional gene silencing, thus regulating multiple biological pathways involved in key cell processes, such as proliferation and differentiation, development, and apoptosis [14].

In this review, we focus on the recent findings linking the alteration of miRNA expression profiles with the exposure to the most common air pollutants (e.g., particulate matter—PM and heavy metals). In particular, we examine the biologic and the prognostic value of miRNAs associable with specific exposures. We also provide an overview of the suitability of miRNA-based research in the complex field of co-exposure to understand the implications of exposures on human health.

Moreover, we review the mechanistic evidence associable with the role of miRNAs in the processes leading to environmental-induced pulmonary disease (i.e., chronic obstructive pulmonary disease COPD) and their potential clinical and prognostic value.

## 2. Air Pollution: The Role Played by Particulate Matter and Heavy Metals

**Figure 1:**
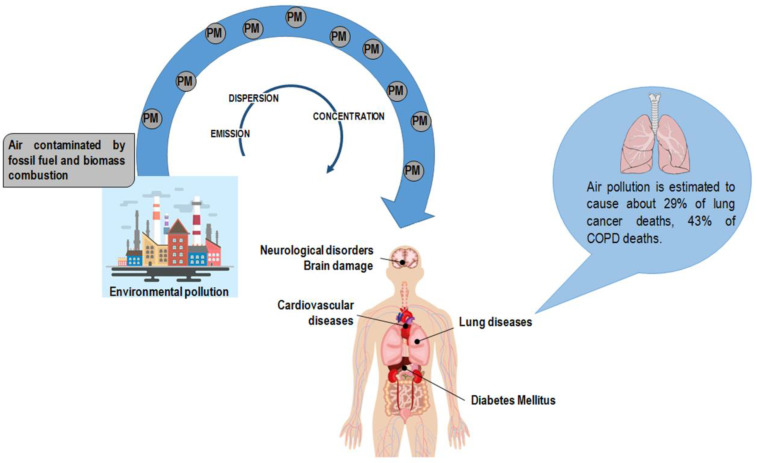
Anthropogenic activity is the primary source of particulate matter (PM) in the atmosphere. PM encompasses a mixture of elements, including arsenic, cadmium, and lead. Air pollution has a deleterious effect on human health exposing the organism to the risk of disease onset. Of note, air pollution is estimated to cause about 29% of lung cancer deaths and 43% of chronic obstructive pulmonary disease (COPD) deaths [19].

Air pollution increases are the primary result of anthropogenic activity derived from industrialization and urbanization and are mainly due to fossil fuel and biomass combustion. Air pollution has a deleterious impact on the environment as it affects groundwater, soil, and air. PM particles of less than 10 and 2.5 μm (PM_10_ and PM_2.5_, respectively) are the leading components of the emissions affecting human health [15]. PM encompasses a mixture of elements categorized as primary particles (i.e., those directly emitted into the atmosphere) and secondary particles (i.e., those resulting from the chemical transformation of gaseous pollutants) [16]. The World Health Organization (WHO) documented the harmful effect of particle pollution (Figure 1) [16,17,18]; humans absorb PM particles, which in turn pass across lung passageways and converge into the bloodstream [19]. Accordingly, PM particles are associated with lung illnesses, including cancer, as well as cardiovascular and cerebrovascular diseases [20,21,22]. It has also to be noticed that the size ranges of PM (0.25, 0.25–0.5, 0.5–1.0, 1.0–2.5, 2.5–10) may be causative of different responses in humans [23]. Scientific and clinical evidence associates the size of particles with the onset and progression of lung disease: the lower the PM, the deeper the penetration into lung tissue, reaching the low respiratory tract [24,25,26].

Heavy metals are widespread pollutants [27,28,29]; their harmful effect is due to their toxicity, persistence in the environment, and bioaccumulative nature [30]. It has been widely acknowledged that these elements are the most important components of PM in the atmosphere with a complex pollution feature [31]. Their presence in ambient air results from anthropogenic activities, such as traffic emissions, industrial processes, and incineration [32]. The most commonly found airborne elements are arsenic (As), cadmium (Cd), and lead (Pb). The European Parliament (Italian transposition Directive: D.Lgs. 155/2010) has fixed the threshold value for their concentration within the atmosphere—As (6ng m^−3^), Cd (5 ng m^−3^), and Pb (500ng m^−3^)—to monitoring and reducing their harmful effects [33].

Arsenic is one of the most abundant elements in nature, and in its inorganic form, it is extremely negative for the environment as well as living creatures. Its anthropogenic generation is mainly due to the use of fossil fuels (e.g., traffic, domestic heating, and natural gas). Contaminated water is the principal way by which humans encounter As [27]. The threshold value given by the WHO for As concentration in drinking water is 10 μg/L [34,35]; a 10–100 fold increase may have a deleterious effect on human health [36]. Airborne levels of As may affect its levels in food and water, thus increasing the route of exposure to this harmful element [37]. Long-term exposure to As puts organisms at risk for cardiovascular diseases, neurological disorders, cancer, pulmonary pathologies, and diabetes mellitus [38]. Disturbance in speech, reduction in locomotor activity, and reduced cognitive performances are described as neurotoxicological effects due to As exposure [39,40]. A study performed on Taiwan high school students evidenced low neurobehavioral performance in subjects from As affected areas compared with those coming from uncontaminated areas [41]. Moreover, the exposure to As causes an increase in the synthesis of ROS, which in turn activates inflammatory response, promotes expression of genes, and deregulates endothelial nitric oxide homeostasis. These processes affect the maintenance of vascular tone [42,43]. As exposure is also associated with a low expression of peroxisome proliferator-activated receptor gamma resulting in a decrease of sensitization of insulin, one of the mechanisms accounting for induction of diabetes mellitus [44,45]. The carcinogenic effect of As has been recognized [46,47]. An in vitro study revealed that even low doses of As induced DNA damage (strand breakage and oxidative stress) [48]. Moreover, As seems to react with thiol groups of zinc binding structure of many transcription factors and protein-controlling cell cycle [49].

Cadmium is the metal of the 20th century because of its widespread use in mineral fertilizers, plastics, batteries, pigments, and metal coatings. Primary non-ferrous metal production and waste incineration are the primary anthropogenic sources of Cd in the air [50,51]. This element persists for a long time in soil and sediments. Its incorporation in fruits and vegetables is significant given the high rate of soil-to-plant transfer. Inhalation is the primary mode of contact between Cd and humans and the principal source of fear for workers and people living in polluted areas. It must be noted that Cd interacts with essential nutrients, thus exerting its harmful effects on the human body [52]. The principal organs affected by Cd exposure are the lung and kidneys. Studies on human and animal models have revealed that Cd exposure leads to osteoporosis, kidney disease, and lung damage. Of note, the International Agency for Research and Cancer has included this element in the Group I list of carcinogens for humans [53].

Lead is a widespread element in the environment because of anthropic activities. Fossil fuel burning, metallurgic industry emissions, and coal combustion emissions are the primary emission sources of Pb. Humans also come in contact with this element in paints, gasoline, cosmetics, toys, and household dusts [54,55]. Pb is extremely dangerous and affects the entire organism. It accumulates in different tissues and organs, causing different diseases ranging from mental retardation, brain damage, autism, and dyslexia to allergies, paralysis, muscular weakness, and kidney damage [27,49,56,57,58,59,60]. Nonetheless, lead toxicity is the primary cause of disease outcome for many organs, and it even causes death [27]. Pb exerts its harmful effect in multiple ways; one is the ability to inhibit calcium actions. Once in the body Pb competes with calcium and interacts with biological molecules causing alterations in their normal actions [49]. Another mechanism accounting for Pb toxicity is its ability to induce oxidative stress. Its effect is mainly exerted on membrane fatty acid composition, thus interfering with processes related to cellular structure such as exocytosis and endocytosis and signal transduction [49]. Pb accumulation negatively impacts on human nervous system by inducing both morphological and pharmaceutical effects [49]. The reduced synthesis of neuronal sialic acid, a molecule that interferes with synapse formation, is considered one of the major morphological changes affecting nervous system [61,62]. Another change is due to early glial cells differentiation [49]. The pharmacological effect of Pb is its substitution for calcium and zinc that leads to neurotoxicity [49]. Khalil and colleagues evidenced in a cohort of exposed and non-exposed workers, that in the former group bone Pb levels predicted lower cognitive performance and cognitive decline over 22 years [63]. Pb has deleterious effects in children, who adsorb this element 4–5 times as much as adult from a given source. It affects their neurodevelopment, causing mental retardation, behavioral disorders, and lowering intelligence quotient [59,60,64]. Lead exposure is also associated to renal diseases. Of note, acute Pb exposition affects the proximal tubular architecture and evokes histological changes consisting in lead–protein complexes and mitochondrial swelling [65]. Two years of Pb exposure causes chronic nephropathy manifested by focal atrophy, loss of proximal tubules, and intestinal fibrosis [56]. Continued exposition to Pb may also have immunotoxic effect and in adults it is associated with sensitization to food allergens [57]. Interestingly, an increase in Pb blood levels of adult subjects is associated with increased sensitization to food allergens, with odds ratio 1.11; 95% confidence interval 1.02 to 1.22 [57].

Despite the diverse detrimental effects exerted on tissue and organs by air pollutants, a common denominator of their adverse effects is the activation of oxidative stress and inflammation [66]. The initiation of the inflammatory response and the generation of reactive oxygen species (ROS) in vascular tissue-evoked chronic exposure to PM particles trigger the etiology of arteriosclerosis [67]. Again, exposure to heavy metals alters the normal balance between pro- and anti-oxidant factors, triggering oxidative stress and predisposing organisms to malignant outcomes [68]. The activation of these processes may trigger pathways and mechanisms, putting organisms at risk for disease onset.

The body of proof concerning the harmful effects of air pollution convinced institutions and the public of the need to limit them. Nevertheless, more studies should address critical issues such as understanding the regulatory mechanisms affected by pollutant exposure, revealing the effects of pollutants on disease risk, and identifying molecular markers for quantification of pollutant toxicity and their prognostic significance.

## 3. The Exposome Concept

Almost a decade ago, Wild introduced the exposome concept to describe all the environmental factors a single individual encounter during a lifetime [69]. This model was extremely fascinating, although its challenges were immediately apparent. According to this model, many variables have to be taken into account, such as the number of pollutants, time-varying exposures, and inter-individual susceptibility [70]. Nevertheless, interest in this approach has increased significantly over time as demonstrated by the large number of articles in PubMed from 2005 to date. This body of literature has helped refine the initial definition of exposome. Recently, Miller and Jones suggested that the exposome concept must include the cumulative measure of environmental influences and the associated biological response through a lifetime. Compared to the first Wild definition, the identification of the “endogenous processes” activated by the body to cope with environmental cues and the concept of biological responses have played a central role in this revised conception [71]. Accordingly, the improved knowledge of the health risks related to pollutant exposures and the mechanisms by which these molecules exert their effect have highlighted the need for exposome and environmental research. This novel definition of exposome will allow researchers to move away from the erroneous concept of “one exposure, one disease” toward a more complex scenario in which both exogenous and endogenous interactions define the organism’s susceptibility to environmental pollutants [5].

In this context, the epigenetic-mediated response to pollutants emerges as a promising framework to examine the multifaceted interactions between the genome and the environment.

## 4. miRNA Response to Environmental Air Pollutants (PMs and Heavy Metals)

During its lifetime an organism struggles with various environmental stressors. The adaptive response to these changes is essential to human survival, and it is associated with phenotypic endpoints [13]. Thus, the analysis of the mechanisms regulating the dynamic changes of gene-mediated pathways following environmental stimulation is an intriguing field of research. The epigenetic mechanisms controlling gene expression seem to be the most recognized explanation of this issue and include DNA methylation, histone modifications, and miRNAs. The hypothesis that the epigenome can be accountable for the phenotypic translation of environmental stimuli through altered gene expression has received more attention over time [72,73]. Franga et al. showed that monozygote twins sharing a common genetic background differed in epigenomic landscape through the aging process. Authors demonstrated that differences in epigenetic patterns could result from the influence of environmental factors (e.g., smoking habits, lifestyle, and diet) [74]. These data pair with the epidemiologic evidence linking exposition to various environmental factors with alterations in epigenetic status [75,76,77,78].

Despite DNA methylation is the most accredited epigenetic mechanism linking the response of the organism to external cues by driving the interactions between genetic and environmental factors, miRNAs have emerged as suitable and robust candidates. This is due to their ability to respond to environmental factors and their critical role in determining phenotypes [79].

MiRNAs are short oligonucleotide sequences (20–24 nucleotides) evolutionarily conserved, consisting of single-stranded non-coding RNA molecules involved in post-transcriptional regulation of mRNAs [80].

MiRNAs have been demonstrated to regulate 30–50% of the mammalian genome through their interaction with mRNAs [81]. The miRNA binding exerts its regulatory action by silencing the expression of target mRNAs. One miRNA can regulate multiple mRNAs since there is no need for a perfect match to activate the silencing machinery. Indeed, a single transcript has multiple binding sites for more miRNAs. This result is a complex regulatory network, giving a new dimension to the intricate mechanisms governing post-transcriptional control [82,83].

Recently, many studies have demonstrated miRNA-expression pattern changes in response to environmental cues [80]; this has increased interest in these molecules as new targets for environmental-based research [84,85]. These molecules fulfil key requirements for biomarkers and/or indicators of environmental-induced tissue injury as well as the mechanisms behind them. First, miRNA expression patterns are organ-, tissue-, or cell-specific, and their modifications may be informative for the evaluation of the toxic effect on multiple tissues or organs. MiRNA expression patterns are also modified by xenobiotic exposure in a time- and dose-dependent manner. These molecules are highly conserved through human and animal models and the experimental settings for their quantitative analyses are robust and standardized. Moreover, miRNA regulatory influence on the mRNA expression profile may be helpful in understanding the adverse outcomes resulting from toxicant exposure [82,85,86]. Finally, miRNAs are present in extracellular fluids, such as serum, plasma, urine, saliva, and cerebrospinal fluid, being encapsulated into extracellular vesicles or associated with carriers, mainly Ago2 or apolipoproteins [87,88].

A decade ago, Izzotti and colleagues provided the first evidence of alterations in miRNA expression induced by environmental exposure. They demonstrated that rats exposed to 28 days of cigarette smoke showed a three-fold downregulation of 24 miRNAs in lung tissues compared with controls. Their data evidenced the alteration of miRNAs, such as let-7, miR-34, and miR-125, regulating lung-cancer related pathways, including stress response, apoptosis, proliferation, and angiogenesis [89]. This seminal work opened an intriguing scenario for environmental-based research. Subsequent studies demonstrated the association between a single exposure to the more widespread contaminants and alterations in miRNA expression patterns (Table 1).

### 4.1. Particulate Matter Exposure and miRNAs

Given the current rapid and global industrialization, particulate air pollution increases have become the most investigated environmental risk factor for human health. This is also true for environmental epigenetics.

In vitro studies have demonstrated that PM exposure influences miRNA expression patterns, driving the development of chronic diseases such as lung cancer, cardiovascular disorders, and respiratory pathologies [103,104,105].

Again, a recent study carried out a global analysis of the circulating levels of miRNAs in the plasma of humans exposed to traffic-related air pollution. Plasma sample was collected from participants who walked for 2 h along the Oxford Street in London. These data have provided useful indications about tissue-specific miRNAs whose alteration was associated with air pollution. Of note, miR-25-3p, miR-30d-5p, and miR-107 were expressed in all target tissue assayed (i.e., lung, heart, brain, breast, kidney, liver, pancreas, and spleen). On the other hand, miR-133a-3p and miR-499a-5p were enriched in the heart, miR-433-3p was highly expressed in the brain, and, finally, miR-1224-5p was pancreas-enriched. These data suggest that air pollution-induced miRNA alterations could influence the physiology of target organs by regulating tissue-specific pathways [90].

Additionally, an in vitro study carried out on lung epithelial cells further demonstrated that non-cytotoxic concentrations of PM_10_, the equivalent dose of 5 days of human exposure, induced a deregulation in the miRNA expression patterns. PM_10_ samples were obtained using a high-volume air sampler (constant flow 1.13 m^3^/min, 3.0 µm cut-off nitrocellulose filters). In particular, miR-8063, miR-4674, miR-6790-5p, miR-1469, and miR-663a were downregulated whereas miR-4448, miR-6808-5p, miR-3147, miR-1298-3p, and miR-125a-3p were upregulated. Bioinformatics analysis predicted that the mRNAs targeted by the downregulated miRNAs were involved in a pathway strictly related to cancer development, such as the wingless/Int (*Wnt*)-, adenylyl cyclase (*cAMP*)-, mammalian target of rapamycin (*mTOR*)-, vascular endothelial growth factor (*VEGF*)-, and adhesion molecular pathways [91]. Rodosthenous and colleagues analyzed the effect of PM_2.5_ on circulating miRNAs in blood samples of healthy subjects. PM values were determined using a hybrid approach combining aerosol optical density with land-use variables together with weather data and PM_2.5_ source emission from monitoring stations. The authors revealed that PM_2.5_ exposure induced an alteration in the expression profiles of seven miRNAs (let-7g-5p, miR-126-3p, miR-130a-3p, miR-146a-5p, miR-150-5p, miR-191-5p, and miR-23a-3p). Further, in silico analysis revealed that potential targets of the identified miRNAs were key mRNAs regulating molecular pathways involved in cardiovascular diseases, including interleukins and growth factors such as platelet-derived growth factor subunit B (*PDGFB*) and vascular adhesion molecule 1 (*VCAM-1*) [92].

A population study also evidenced the changes in miRNA expression patterns in young subjects living in severe particulate air pollution sites. Indoor PM_2.5_ were measured by personal exposure monitors while outdoor ones were monitored with GRIMM EDM180 dust monitor. An inverse association between miRNAs expression and PM_2.5_ exposure was demonstrated. Interestingly, a paired analysis of the expression levels of miRNAs and those of their known target mRNAs revealed potential associations linking circulating miRNA and the modifications of genes regulating the immune responses vasoconstriction and coagulation. Specifically, an inverse association among the circulating levels of five miRNAs (miR-21-5p, miR-187-3p, miR-146a-5p, miR-1-3p, and miR-199a-5p) and their potential targets, such as interleukin-1, tumor necrosis factor, Toll-like receptor-2, and endothelin 1, has emerged [93].

### 4.2. Arsenic Exposure and miRNAs

Chen and colleagues investigated the effect induced by As on human L-02, a normal liver cell line. They demonstrated that arsenic exposure induced a malignant transformation in L-02 cells associated with the overexpression of a specific miRNA, miR-191. Inhibition of miR-191 in L-02 transformed cells resulted in reduction of malignant properties, such as the activation of the epithelial to mesenchymal transition and the formation of cancer stem cells. These findings indicate that miR-191 is a useful biomarker for malignancy processes related to As exposure [94].

Additionally, an in vivo study on rats exposed to different concentrations of sodium arsenite (0, 0.1, 1, 10 and 100 mg/L) for 60 days indicated a concentration-dependent alteration of miRNA expression profiles. Five miRNAs showed a significant concentration-dependent deregulation. In particular, two out of five miRNAs, miR-151 and miR-183, were overexpressed following As exposure. On the other hand, the last three miRNAs, miR-26a, miR-423, and miR-148b, resulted in downregulation. Among them, several miRNAs targeted antioxidant mRNAs, including glutamate–cysteine ligase (*Gcl*), catalytic subunit (*Gclc*), and modifier subunit (*Gclm*), which are involved in glutathione (*Gsh*) synthesis. The effect of As on miRNA expression were mainly observed in the high concentration groups. These findings support the hypothesis that oxidative stress is a possible mechanism of action associated with As exposure [95]. A recent population study conducted on a cohort of Mexican children exposed to inorganic As showed alterations in miR-126 expression profiles. In particular, authors evidenced a significant negative association between As urinary concentration and miR-126 plasmatic levels. Experimental findings, linking miR-126 downregulation to the likelihood of suffering from cardiovascular illnesses, have suggested mechanistic speculations [96].

### 4.3. Cadmium Exposure and miRNAs

Normal human prostate cells (RWPE-1) exposed to Cd undergo malignant transformation in vitro and, once inoculated in mice, induce highly invasive adenocarcinoma. Ngalame and colleagues demonstrated an alteration of miRNA expression profiles in Cd-transformed RWPE-1 such as miR-155, miR-205, and miR-134 [97]. Bioinformatics analysis identified the potential targets involved in oncogenesis and cancer progression, and their expression levels were further analyzed. Of note, the authors observed an increase in the mRNA levels of the oncogene Kirsten rat sarcoma viral oncogene homologue (*KRAS*) and the E2F transcription factor 1 (*E2F1*) gene involved in cell signaling [97]. Interestingly, similarities between Cd- and As-transformed prostate cells were observed in terms of deregulated patterns of miRNAs. For example, upregulation of miR-96 and miR-9 was common in both transformed cells as well as downregulation of miR-205, miR-155, miR-373, miR-138, and miR-222 [97]. An in vivo study carried out on a rat model of Cd-induced kidney injury provided further evidence demonstrating miRNA alteration following metal exposure. Microarray results revealed that 98 miRNAs were deregulated in renal cortex of exposed rats compared with healthy controls. Of those, 44 were upregulated (including miR-21-5p, miR-34a-5p, miR-146b-5p, miR-149-3p, miR-224-5p, and miR-451-5p) and 54 were downregulated (including miR-193b-3p, miR-455-3p, and miR-342-3p). These findings suggest that Cd-induced miRNA dysregulation might play a role in nephrotoxic phenotypes. Further, the authors speculated on the suitability of these molecules as biomarkers for kidney sufferance induced by pollutant exposure [99].

As the major component of cigarette smoke, Cd was investigated for its induced alterations in lung tissue. Hassan and colleagues showed that Cd and cigarette smoke induced the upregulation of two miRNAs, miR-101 and miR-144, in lung epithelial cells. They demonstrated that the two molecules targeted the cystic fibrosis transmembrane conductance regulator (*CFTR*) gene and downregulated its expression levels in airway epithelial cells in vitro. *CFTR* is a commonly expressed gene involved in the control of airway surface fluid homeostasis. Of note, patients suffering from chronic obstructive pulmonary disease (COPD) evidenced an increase of miR-101 expression; thus, the authors suggested a possible connection between miRNA deregulation, Cd exposure, and pathophysiological implications [98,106].

Epidemiologic investigations also demonstrated that Cd-exposure resulted in alteration of circulating miRNA [107]. In particular, a study performed on steel workers exposed for three days to PM containing Cd revealed an increase in miR-146a levels in peripheral blood leukocytes. These data provided mechanistic insights into the gene network leading to Cd-induced increases of cancer and cardiovascular diseases [100].

### 4.4. Lead Exposure and miRNAs

Unlike the other metals, the experimental evidence associating Pb exposure to miRNA deregulations is sparse [107]. An and colleagues first reported the effect of Pb exposure on miRNA expression. Microarray analysis revealed that chronic lead exposure induced a different expression pattern of seven miRNAs in rat hippocampus: miR-204, miR-211, miR-448, miR-449a, miR-34b, and miR-34c. Bioinformatics analysis evidenced the gene pathways regulated by the altered miRNAs, resulting in neurodegeneration, neural injury, and neural regeneration. This evidence allowed associating Pb-induced miRNA deregulation with alteration of neurophysiological pathways and risk of neurodegenerative disease [101]. Recently, Xu and colleagues carried out a pivotal study in which they studied the expression pattern of plasma miRNAs in workers exposed to Pb. Their data demonstrated the susceptibility of circulating miRNAs following Pb exposure. Indeed, microarray analysis and qRT-PCR validation provided a signature consisting of one upregulated miRNA (miR-572) and three downregulated miRNAs (miR-520c-3p, miR-211, and miR-148a). Besides the significance for biomarkers-based research, these data pave the way for further targeted analysis of the pathways regulated by these molecules and may provide useful insights to understand Pb-induced health outcomes [102].

These findings helped to focus the attention of the scientific community on miRNAs as suitable tools for understanding the mechanisms behind environmental epigenetics and the implications of toxicants on health outcomes. Furthermore, miRNA expression profiles may provide a “fingerprint” reflecting environmental exposure cues. The effect of environmental exposure on miRNA expression profiles and on regulatory mechanisms driven by these molecules provide promising and fascinating possibilities. This evidence also suggests the versatile and informative nature of miRNAs for developing more effective prevention strategies for environmental diseases.

## 5. Co-Exposure Strategy in Environmental-Based Research and the Contribution of miRNAs

The epigenetic marks induced by lifetime exposure to environmental influences may act as a “fingerprint” of the exposure and/or may provide useful insights for ecological and clinical research [13]. The findings reported in this review demonstrate the effect of environmental pollutants on the deregulation of miRNA expression patterns and how they may affect several mechanisms associated with the susceptibility of different diseases [108]. Several studies have elucidated the exposure-related risk to global health [72,103,109,110]. This has allowed investigators to address difficulties and challenges that need to be overcome. In this section, we provide an overview of co-exposure significance as the principal drawback of environmental-based strategies.

Most toxicology researchers have focused on the effects induced by a single exposure to a particular pollutant. Nevertheless, humans are exposed to a mixture of toxic substances at low doses. Experimental evidence showed that metal mixtures (including Cd and Pb) induced many types of genotoxic effects on red blood cells, bone marrow, and spleen cells in different animal models [111,112]. In particular, researchers observed the induction of apoptosis, chromosomal aberrations, and oxidative stress-caused DNA damage [111,112]. Other findings proved the effect of a Pb-As-Cd in ROS production affecting the kidneys, brains, and livers of rats exposed to metal blended enriched water [29,113,114,115,116]. Analogously, metal mixtures also showed a deleterious influence on the immune and neural systems. The combined action of several metals, including As, Pb, and Cd, resulted in immunotoxicity for rats that experienced deficiency in cell-mediated immune responses [29,116]. Moreover, metal mixtures have been demonstrated to be detrimental for brain integrity. Rats exposed to sub-chronic levels of an As-Pb-Cd blend experienced a neuronal developmental disorder evoked by the synergistic actions of these chemicals. These findings fueled the hypothesis about the ability of metal mixtures to pass the blood-brain barrier, thus raising the risk of cognitive dysfunction for exposed individuals [117].

Despite this evidence describing the risk associated with metal mixtures, the mechanisms of action activated by the synergistic conduct of these toxicants are still unclear. Thus, an effective environmental-based study must consider the epigenetic perturbations evoked by a mixture of pollutants. This strategy is in line with the paradigm-shift concept defining the exposome as the cumulative measure of environmental influences and the associated biological response [71,118].

In 2016, Ji and colleagues studied the relationship between co-exposure to environmental contaminants and alterations in miRNA expression profiles. Their experimental hypothesis was due to evidence revealing the key role of miRNAs in regulating biological and physiological responses to air pollution in lung tissue. The authors aimed to demonstrate that a miRNA-targeted gene expression might be accountable for the functional impairment of lung tissue leading to pulmonary hypertension following a combined exposure to air pollutants. Thus, they treated C57BL/6 mice with different concentrations of a mixture mimicking the combined exposure of coal-burning air-pollution (SO_2_, NO_2_, and PM_2.5_). Their data revealed a co-exposure-induced alteration in the miR-338-5p expression profile. Of note, the downregulation of this miRNA resulted in a stimulated expression of its target gene *Hif1α*, a key player in the *Hif1α /Fhl-1* pathway. These data suggested that co-exposure to air pollutants induced a miR-338-5p-mediated alteration of the *Hif1α /Fhl-1* pathway, predisposing mice to pulmonary hypertension [119].

Martínez-Pacheco and colleagues provided evidence about the changes in the miRNA expression pattern in fibroblast exposed to 4-hr treatments with a metal mixture (consisting of As, Cu, and Pb). The authors described the overexpression of seven miRNAs (miR-10, miR-133, miR-154, miR-204, miR-222, miR-375, and miR-379), which are involved in key processes such as inflammatory response, cell death, cell growth and proliferation, and cancer. Indeed, downregulation of 42 predicted target mRNAs involved in the above processes further validated their results. Overall, these data have provided an epigenetic-based mechanistic model establishing the health effect of metal mixtures at epidemiologically relevant concentrations [120]. Deng et al. provided further data associating miRNA deregulation induced by exposure to combined pollutants with early health damage, including oxidative stress, genetic damage, and cardiac alterations. In particular, they analyzed the combinatory effect of metals and aromatic hydrocarbons on 360 healthy men, revealing that the complex interaction among these pollutants affected the expression profile of miRNAs. This effect could be associated with early health damage, providing evidence about the prognostic and the mechanistic value of the miRNA signature [121].

This evidence demonstrates that the co-exposure approach may provide a comprehensive overview of the complex interactions among environmental epigenetics and human health. In this context, miRNA-based research could be the gold standard to provide suitable prognostic markers as well as useful insights for determining the underlying mechanisms triggering environmental-induced diseases. Indeed, miRNAs are newly emerged as gene expression regulatory factors that may easily translate the evidence resulting from exposure to pollutant mixtures into prognostic and preventive strategies.

## 6. Air Pollution and COPD: The Role of miRNAs

As discussed in previous paragraphs, PM particles are the principal components of air pollution, and much evidence indicates that their inhalation predisposes humans to a range of adverse respiratory health outcomes [122]. PM particles elicit an immune response and increase oxidative stress, and in addition, to the harmful consequences on the respiratory apparatus, they may function as adjuvants exacerbating the effects induced by allergens or respiratory viruses [123].

Epidemiologic data showed the influence of particulate air pollution on COPD, which is one of the major causes of death for both genders worldwide (3.2 million deaths/year) [124,125]. COPD encompasses bronchial asthma, chronic bronchitis, and pulmonary emphysema and is characterized by a progressive and irreversibly poor airflow. It is described as a multifactorial disease triggered by both genetic and environmental risk factors [126]. The correlation between the rate of decline of COPD patients and the level of their exposure to air pollution was evidenced in 1993 by Pope and Kanner [127]. These data were supported by other epidemiologic evidences over the years; thus, air pollution is now considered as a risk for COPD exacerbation [119]. Air pollution seems to trigger the same pathophysiological effects described for cigarette smoke, which is the best documented COPD-risk factor. The principal mechanisms involved in the pathogenesis of COPD seems to be the insurgence of chronic airway inflammation, induction of oxidative stress, and imbalance of cellular homoeostasis in lung tissue [126,128]. Nevertheless, air pollution more severely involves the bronchi, induces great oxygen desaturation, and significantly impacts a person’s quality of life [129].

Recent evidence assessed the role played by PM in the principal mechanisms leading to COPD. Li and colleagues demonstrated that acute exposure to PM_2.5_ evoked an increased inflammatory response with a high infiltrative rate and an increased number of inflammatory cells in the broncho-alveolar lavage fluid [130]. Moreover, particulates, once inhaled, generate free radicals, affecting the lung cells and triggering oxidative damage. This effect could be enhanced by the metal components carried by PM particles, which, in turn, increase the production of ROS in cells [131]. Of note, epigenetic modifications in lung tissue following PM exposure have been evidenced. Chen and colleagues designed an intriguing study on COPD patients in Shanghai that revealed a correlation between PM_2.5_ and DNA methylation regulating exhaled nitric oxide (FeNO). FeNo is a marker for airway inflammation, and its deregulation-induced particulate exposure seemed to aggravate the inflammatory status of COPD patients [93].

Despite the body of literature, a deep knowledge of both the pathogenic mechanisms driving COPD and prognostic factors is still poor. Indeed, the risk of underdiagnosis is one of the principal issues in COPD epidemiology. On these premises, the need for future studies dissecting the pathogenesis of COPD and arguing diagnostic and protecting strategies are crucial, especially considering the increase in air pollution. MiRNAs seem to fully address these issues because, by their innate nature, they serve as both biomarkers and therapeutic targets. These molecules, by acting at post-transcriptional levels, are involved in the regulation of many biological processes, even those closely related to respiratory injuries [132]. As reviewed in the preceding paragraphs, their expression profile is strictly susceptible to air pollutants. Therefore, considering the heterogenic composition of air pollution and the consequent co-exposure to different toxicants, miRNAs could be intriguing candidates to assess gene expression patterns inducing functional impairment in lung tissue.

An in vitro study on human bronchial epithelial cells (HBECs) provided evidence for the regulatory networking enhancing inflammatory response following PM_2.5_ exposure and its implication for COPD. The authors demonstrated the particulate-induced upregulation of a long non-coding RNA (lncRNA RP11-86H7.1), which, in turn, acts as a sponge for miR-9-5p. The sequestration of this miRNA impaired its negative regulatory function on the nuclear factor kappa B subunit 1 (*NFKB1*) gene and sustained the activation of nuclear factor kappa-light-chain-enhancer of activated B cells (*NF-kB*) signaling. This led to an enhanced expression of inflammatory factors promoting PM-induced airway inflammation [128]. Indeed, other evidence showed the regulative role played by miR-9-5p, via targeting the *NFKB1* gene, in reducing the expression levels of such pro-inflammatory genes such as tumor necrosis factor alpha, interferon gamma, and interleukin 6 [133]. This study poses intriguing mechanistic and prognostic evidence regarding airway inflammatory diseases, such as COPD, caused by PM_2.5_ exposure.

An epidemiologic study carried out in a cohort of mother–newborn pairs was set up to investigate the potential alterations of miRNA expression profiles and their target genes in placental tissue induced by parental exposure to particulate air pollution [134]. The authors showed that PM_2.5_ induced an alteration in the expression profiles of two miRNAs, miR-222 and miR-21, with a concomitant increase of the common putative target gene phosphatase and tensin homolog (*PTEN*). Interestingly, *PTEN*, which negatively regulates the PI3K/AKT pathway, modulates many aspects of cell biology, such as survival and cycle progression, and is described as a predisposing gene of COPD. This evidence led the authors to speculate that in-utero exposure to particulate pollution may trigger changes in miRNA expression profiles, potentially predisposing a person to pulmonary outcomes in later life [126,134].

In 2018, Zhou and colleagues carried out an interesting study in which they screened COPD- associated miRNAs to detect those differentially expressed and then observed their trends during PM_2.5_ exposure. The authors identified 21 differentially expressed miRNAs in COPD patients with respect to control subjects; among them, their attention focused on miR-495-3p, miR-223-5p, and miR-194-3p. Interestingly, miR-194-3p seemed to be involved in secretion of the extracellular matrix during fibrosis, and miR-223-5p was upregulated in the lung tissue of smokers suffering from COPD. The expression profiles of these miRNAs were validated in a time-series study, which showed a PM_2.5_-induced decrease and an increase in the blood levels of miR-194-3p and -223-5p, respectively. Of note, miR-194-3p highly correlated with lung ventilation function following particulate exposure [135]. Overall, these data provide intriguing insights into the potential role of miRNA in understanding the link between environmental exposure and COPD as well as providing prognostic evidence.

Given the role of heavy metals in driving lung inflammation and dysfunction with their accumulation, the significance of Cd in regulating a miRNA response upon exposure and its association with COPD was tested. Recently, an interesting study screened the serum samples of COPD patients and identified downregulation of miR-181a-2-3p. This miRNA played a role in regulating an inflammatory response in human epithelial cells in bronchi. It is described as a cytokine-responsive miRNA, which drives cellular response to inflammation. Of note, in vitro studies demonstrated that the expression levels of miR-181a-2-3p were also downregulated in Cd-treated bronchial epithelial cells. This evidence suggested a miRNA role in inflammatory responses, leading to COPD. Indeed, the silencing of miR-181a-2-3p in Cd-treated cells showed an increase in inflammatory response. Global gene expression profiling allowed the identification of miR-181a-2-3p targets involved in Cd-induced inflammatory responses in human bronchial epithelial cells. In particular, the authors demonstrated the involvement of the Toll-like receptor 4 (*TLR4*), which is a fine sensor influencing inflammatory cascade by stimulation of pro-inflammatory molecules [136,137]. These data evidenced the role of miR-181a-2-3p and its target *TLR4* in the regulation of inflammatory response in bronchial cells upon Cd exposure and their potential role in COPD pathophysiology. Bollati and colleagues evaluated the effect induced by metal-rich PMs in the expression profiles of miRNAs involved in regulation of inflammation and oxidative stress. The blood samples of electric furnace steel plant workers revealed an increased expression of miR-222 and miR-21 with an interesting association of the former with Pb exposure. Moreover, the authors detected an association between exposure to Cd and Pb and miR-146a, although the miRNA expression levels did not show a statistically significant difference in the control samples. Of note, the increased plasma levels of miR-222 let authors hypothesize a metal particulate-induced increase of inflammatory response [100].

**Figure 2:**
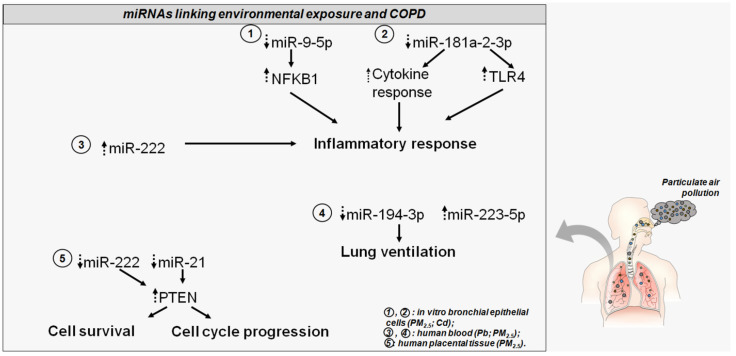
The altered miRNAs disturb COPD-related genes and pathways. Dotted head-up arrows indicate upregulated miRNAs/genes due to pollutant exposure; dotted head-down arrows indicate downregulated miRNAs/genes due to pollutant exposure. Circled numbers in the bottom right legend illustrate, for each miRNA, the pollutant exposure, and the source of miRNA detection.

Overall, this evidence leads to an appreciation of the suitability of miRNAs in understanding the regulatory mechanisms induced by particulate exposure and predisposition to COPD as well as the potential clinical and prognostic value of these molecules (Figure 2). This could address the adaptive response to environmental cues from air pollution and might provide further insights regarding new prevention and treatment strategies for airway inflammatory diseases, such as COPD.

## 7. Conclusions and Future Perspectives

Worldwide attention has increased regarding the levels of environmental pollution affecting different aspects of the human body. Environmental epigenetics has emerged as a promising tool for understanding and marking the diverse and detrimental impacts of air pollution exposure.

Epigenetic signatures may provide prognostic insights for detecting exposures characterizing a particular community or area. On the other hand, the increased understanding of the effects exerted by environmental factors on miRNAs relating to disease outcomes and to their use as targets for preventive strategies has a powerful translational significance.

The growing extensive lists of miRNAs responsive to environmental stimuli should be integrated with the mechanistic evidence behind these changes. This evidence may uncover whether miRNA alterations are the drivers or the symptoms of the (patho) physiological processes an organism undergoes in response to exposure [83]. Again, co-exposure-based strategies may reveal the interactions among the different toxins comprising air pollutant mixtures. Intriguing findings may describe the differences in the responses activated by the most studied pollutants when assayed individually or as a mixture. Improved knowledge of health risks from the combined effect of environmental toxicants, exposure time, and levels is critical for future environmental research. In this scenario, miRNAs have emerged as suitable candidates to determine pathophysiological alterations due to single or combined exposure to air pollutants.

At present, little is known about the long-term effects of environmental exposure on epigenome changes. To address this issue, targeted interventional studies need to be developed.

Finally, the increasing evidence regarding the role played by miRNAs in driving lung tissue responses to particulate air pollution should be investigated by tailored population studies. These findings could be pivotal for understanding the mechanistic pathways regulating the alterations induced by environmental exposure in lung tissue and predisposing a person to severe pulmonary disease such as COPD. Further findings may also provide insights into the targeted populations susceptible to airway dysfunctions.

## Figures and Tables

**Table 1:** The effect of pollutant exposure on miRNA expression profiles and its implications.

Chemical/Source	Model	miRNA Modification	Effect	Reference
Particulate matter (PM_10_; PM_2.5_)	In vivo	↑ miR-25-3p, ↑miR-30d-5p and ↑miR-107	Systemic biomarkers for traffic-related air pollution	[90]
		↓ miR133a-3p, ↓miR-499a-5p	Biomarkers for traffic-related air pollution effect in heart	
		↓ miR-433-3p	Biomarkers for traffic-related air pollution effect in brain	
		↑ miR-1224-5p	Biomarkers for traffic-related air pollution effect in pancreas	
	In vitro	↑ miR-4448, ↑miR-6808-5p, ↑miR-3147,↑ miR-1298-3p and ↑miR-125a-3p	Regulatory effect on pathway strictly related to cancer development	[91]
	In vivo	↓ miR-8063, ↓miR-4674, ↓miR-6790-5p, ↓miR-1469 and ↓miR-663a	Regulation of molecular pathways involved in cardiovascular diseases	[92]
		↑ let-7g-5p, ↑miR-126-3p, ↑miR-130a-3p,↑miR-146a-5p,↑miR-150-5p, ↑miR-191-5p, and ↑miR-23a-3p.		
	In vivo	↓ miR-21-5p, ↓miR-187-3p, ↓miR-146a-5p, ↓miR-1-3p and ↓miR-199a-5p	Modifications of genes regulating immune response, vasoconstriction and coagulation	[93]
Arsenic	In vitro	↑ miR-191	Malignant transformation of L-02 cells	[94]
	In vivo	↑miR-151;↑miR-183	Oxidative stress	[95]
		↓ miR26a, ↓miR-423 and ↓miR-148b		
	In vivo	↓ miR-126	Increased risk for cardiovascular illnesses	[96]
Cadmium	In vitro	↑ miR-96 and ↑miR-9	Malignant transformation of RWPE-1cells	[97]
		↓ miR-205, ↓miR-155, ↓miR-373, ↓miR-138 ↓miR-222 and ↑miR-134		
	In vitro	↑miR101 and↑ miR-144	Increased risk of pulmonary disease (e.g.,COPD)	[98]
	In vivo	↑ miR-21-5p, ↑miR-34a-5p, ↑miR-146b-5p, ↑miR-149-3p, ↑miR-224-5p and ↑miR-451-5p	Nephrotoxic phenotype	[99]
		↓ miR-193b-3p, ↓miR-455-3p, and ↓miR-342-3p		
	In vivo	↑ miR-146a	Increased risk for cancer and cardiovascular diseases	[100]
Lead	In vivo	↑ miR-204, ↑ miR-211, ↑miR-448, ↑miR-449a, ↑miR-34b, and ↑miR-34	alteration of neurophysiological pathways and risk of neurodegenerative disease	[101]
	In vivo	↑ miR-572	Systemic biomarkers for Pb-exposure	[102]
		↓ miR-520c-3p, ↓miR-211, and ↓miR-148a		

↑= increase; ↓= decrease.

## Abbreviations

As	Arsenic
cAMP	Adenylyl cyclase
Cd	Cadmium
CFTR	Cystic Fibrosis Transmembrane Conductance Regulator
COPD	Chronic Obstructive Pulmonary Disease
E2F1	E2F transcription factor 1
GCL	Glutamate–Cysteine Ligase
GCLC	Glutamate–Cysteine Ligase Catalytic Subunit
GCLM	Glutamate–Cysteine Ligase Modifier Subunit
GSH	Glutathione
HBECs	Human Bronchial Epithelial Cells
KRAS	Kirsten rat sarcoma viral oncogene homologue
Pb	Lead
lncRNA	long non-coding RNA
mTOR	Mammalian target of rapamycin
miRNAs	Micro-RNAs
RWPE-1	Normal human prostate cells
NFKB1	Nuclear Factor Kappa B Subunit 1
NF-kB	Nuclear Factor Kappa-Light-Chain-Enhancer Of Activated B Cells
PM	Particulate matter
PTEN	Phosphatase and Tensin Homolog
PDGFB	Platelet-Derived Growth Factor Subunit B
ROS	Reactive Oxygen Species
RISC	RNA-induced silencing complex
WHO	The World Health Organization
TLR4	Toll-Like Receptor 4
VEGF	Vascular Endothelial Growth Factor
VCAM-1	Vascular adhesion molecule 1
Wnt	Wingless/Int
